# GPR84-mediated signal transduction affects metabolic function by promoting brown adipocyte activity

**DOI:** 10.1172/JCI168992

**Published:** 2023-12-15

**Authors:** Xue-Nan Sun, Yu A. An, Vivian A. Paschoal, Camila O. de Souza, May-yun Wang, Lavanya Vishvanath, Lorena M.A. Bueno, Ayanna S. Cobb, Joseph A. Nieto Carrion, Madison E. Ibe, Chao Li, Harrison A. Kidd, Shiuhwei Chen, Wenhong Li, Rana K. Gupta, Da Young Oh

**Affiliations:** 1 Touchstone Diabetes Center, Department of Internal Medicine, University of Texas Southwestern Medical Center, Dallas, Texas, USA.; 2 Department of Anesthesiology, McGovern Medical School, University of Texas Health Science Center, Houston, Texas, USA.; 3 Division of Endocrinology, Department of Medicine, Duke Molecular Physiology Institute, Durham, North Carolina, USA.; 4 Department of Cell Biology, University of Texas Southwestern Medical Center, Dallas, Texas, USA.

**Keywords:** Cell Biology, Metabolism, Adipose tissue, Calcium, Molecular biology

## Abstract

The G protein–coupled receptor 84 (GPR84), a medium-chain fatty acid receptor, has garnered attention because of its potential involvement in a range of metabolic conditions. However, the precise mechanisms underlying this effect remain elusive. Our study has shed light on the pivotal role of GPR84, revealing its robust expression and functional significance within brown adipose tissue (BAT). Mice lacking GPR84 exhibited increased lipid accumulation in BAT, rendering them more susceptible to cold exposure and displaying reduced BAT activity compared with their WT counterparts. Our in vitro experiments with primary brown adipocytes from GPR84-KO mice revealed diminished expression of thermogenic genes and reduced O_2_ consumption. Furthermore, the application of the GPR84 agonist 6-*n*-octylaminouracil (6-OAU) counteracted these effects, effectively reinstating the brown adipocyte activity. These compelling in vivo and in vitro findings converge to highlight mitochondrial dysfunction as the primary cause of BAT anomalies in GPR84-KO mice. The activation of GPR84 induced an increase in intracellular Ca^2+^ levels, which intricately influenced mitochondrial respiration. By modulating mitochondrial Ca^2+^ levels and respiration, GPR84 acts as a potent molecule involved in BAT activity. These findings suggest that GPR84 is a potential therapeutic target for invigorating BAT and ameliorating metabolic disorders.

## Graphical abstract

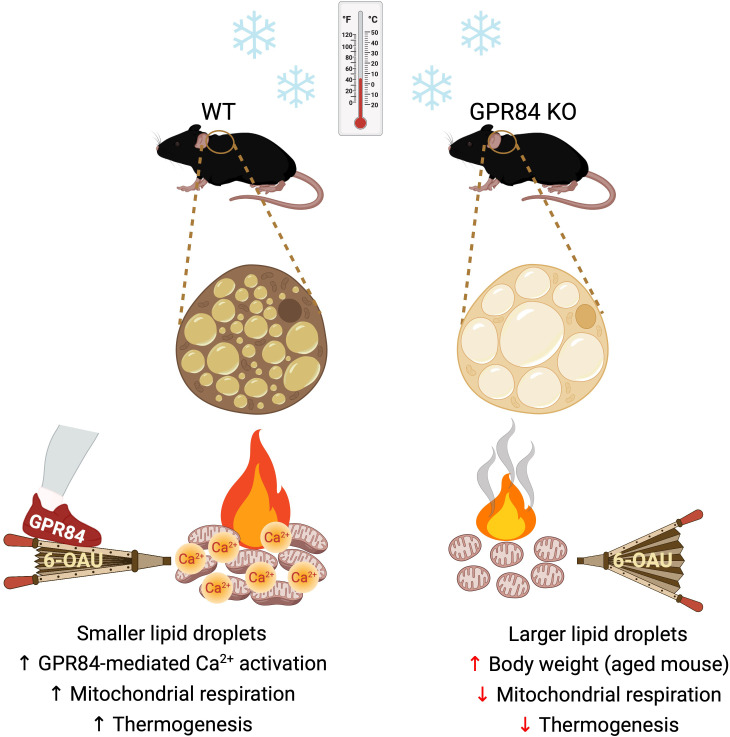

## Introduction

Adipose depots have attracted considerable interest because of their pivotal roles in orchestrating systematic energy homeostasis (1, 2). Within this intricate network, brown adipose tissue (BAT) has emerged as a distinctive player with the capacity to enhance energy expenditure by dissipating chemical energy (3). The intricate equilibrium maintained by BAT extends beyond its thermogenic capacity, and disruptions therein have been unequivocally linked to perturbations in both body temperature regulation and glucose homeostasis (4). Consequently, unraveling the intricate pathophysiological conditions that impinge upon adipose tissue, particularly BAT, holds promise for unveiling novel therapeutic approaches for tackling metabolic disorders.

Increasing BAT activation is a promising strategy against storing excess energy (5–7). Functioning as a thermogenic hub, BAT helps maintain the core body temperature of mammals that navigate cold environments (8). In humans, BAT comprises stromal tissue, white adipose tissue (WAT), and thermogenic adipocytes that contain uncoupling protein-1 (UCP1) (9). Upon activation, BAT takes up fatty acids (FAs) and glucose to catalyze heat generation (10) to ensure homeostasis of core body temperature. Augmenting this thermal orchestration, beige/brite thermogenic adipocytes mobilize within WAT in response to stimuli such as cold exposure and β_3_-adrenergic receptor agonists (11, 12). BAT activation not only culminates in the regulation of body temperature, but also bolsters whole-body glucose homeostasis and insulin sensitivity in both humans and mice following cold exposure (13, 14). Thermogenesis in brown adipocytes predominantly depends on the mitochondria. Mitochondrial dysfunction in BAT leads to metabolic diseases, such as insulin resistance, dyslipidemia, and impaired thermogenesis (12, 15, 16). Therefore, BAT activation is a promising target for the treatment of metabolic disorders.

G protein–coupled receptors (GPCRs) play critical roles in the regulation of physiological and pathological processes, and represent a class of important drug targets. Free fatty acids (FFAs) act as ligands for multiple GPCRs (17). For example, GPR41 and GPR43 are activated by short-chain FAs (<C6), resulting in the inhibition of cAMP production through the G_i/o_ signaling pathway (18, 19). Long-chain FAs (>C12) bind to GPR40, eliciting an elevation in intracellular Ca^2+^ levels through the G_q/11_ signaling pathway, which in turn leads to the stimulation of insulin secretion by pancreatic β cells (20, 21). GPR120 is also activated by long-chain polyunsaturated FAs, resulting in the activation of both G_q/11_ and β-arrestin-2–mediated activation of the ERK and PI3K/AKT pathways, which improves insulin sensitivity and inhibits inflammation (22–25).

G protein–coupled receptor 84 (GPR84) is a medium-chain FA receptor whose function is largely unknown, except in macrophages (26, 27). Although GPR84 is highly expressed in immune cells and is involved in regulating the inflammatory response in macrophages, its precise role in regulating inflammation remains uncertain (27–30). Medium-chain FA levels change under various metabolic disturbances such as starvation (31), feeding (32), cold exposure, type 1 diabetes (33), and obesity, indicating that GPR84 activity may vary under these conditions (31–34). In addition, GPR84 influences lipid metabolism in metabolic diseases (29). However, the specific role of GPR84 under these metabolic conditions remains unclear. In this study, we report that GPR84 is highly expressed in BAT and that its expression is upregulated by cold stimulation. Therefore, we investigated the role of GPR84 in BAT activation during cold exposure, its intracellular signaling following GPR84 stimulation in brown adipocytes, and its impact on mitochondrial function, revealing the intricate interplay between GPR84 and these physiological determinants.

## Results

### GPR84 is highly expressed in brown adipocytes.

**Figure 1:**
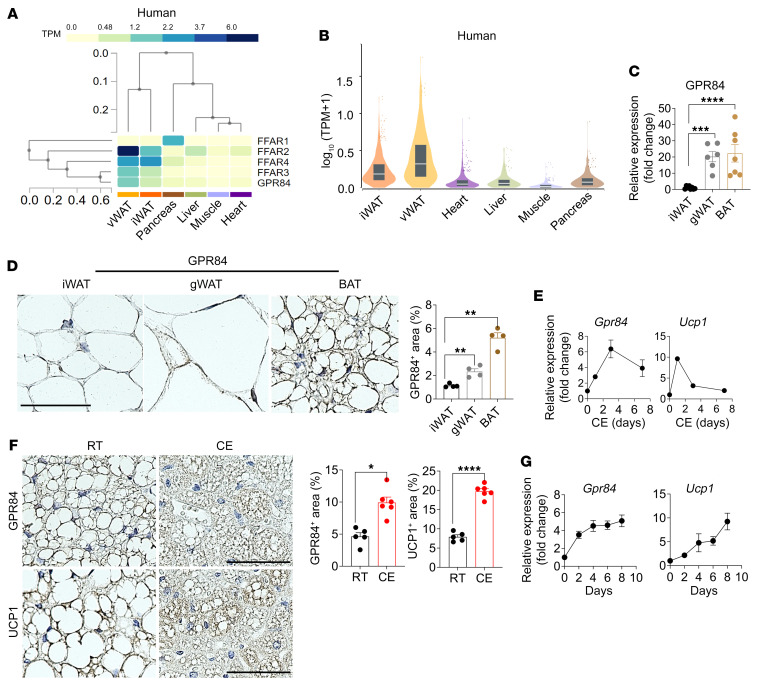
GPR84 expression in BAT. (**A**) Expression of different FA receptors in metabolic tissues in human samples from the GTEx database. vWAT, visceral WAT. (**B**) Expression of GPR84 in different metabolic tissues in human samples from the GTEx database. Bulk tissue gene expression level of GPR84 (Data Source: GTEx Analysis Release V8; ENSG00000139572.3). (**C**) GPR84 mRNA expression levels were measured by qPCR in different adipose tissues. *n* = 6–7/group. (**D**) Representative images of GPR84 IHC staining in different mouse adipose tissues. Data are representative of more than 5 images from at least 3 independent mouse cohorts. Scale bar: 50 μm. Data in bar graphs of the GPR84^+^ area are represented as means ± SEM. *n* = 4/group/cohort. (**E**) GPR84 and UCP1 mRNA expression were measured by qPCR in WT mouse BAT during cold exposure (CE). *n* = 3/each time point. (**F**) IHC images of GPR84 and UCP1 in BAT of WT mice exposed to cold and RT. Scale bars: 50 μm. Bar graph indicates quantification of the GPR84^+^ and UCP1^+^ areas analyzed by ImageJ from more than 3 images as means ± SEM from at least 3 independent mouse cohorts. *n* = 5–6/group/cohort. (**G**) GPR84 and UCP1 mRNA expression were measured by qPCR during brown adipocyte differentiation. Data are represented as means ± SEM of at least 3 independent experiments in triplicate. *P < 0.05; **P < 0.01; ***P < 0.001; ****P < 0.0001, 2-tailed Student’s *t* test (**F**); 2-way ANOVA followed by Bonferroni’s multiple-comparison test (**C** and **D**). See also Supplemental Figure 1. Primer sequences are available in Supplemental Table 1.

We initially quantified the expression of GPR41, GPR43, GPR120, and GPR84 in various human tissues. Notably, the adipose tissue exhibited significantly elevated expression levels of these FFA receptors compared with other metabolically active tissues, such as the pancreas, liver, and muscles (Figure 1, A and B). Furthermore, a similar pattern of expression was observed for GPR84 and UCP1 in human adipose tissue (Supplemental Figure 1A; supplemental material available online with this article; https://doi.org/10.1172/JCI168992DS1). Although the functions of GPR43 (FFAR2) (35), GPR120 (FFAR4) (36), and GPR41 (FFAR3) (37) in the adipose tissue have been relatively well elucidated, the precise role of GPR84 in adipose tissue remains unclear. Therefore, we validated the GPR84 expression profiles across various tissues, including distinct adipose depots, in both humans (Figure 1B and Supplemental Figure 1A) and mice (Figure 1C and Supplemental Figure 1B). GPR84 demonstrated robust expression in both human and mouse BATs (Supplemental Figure 1, A, C, and D), indicating their potential significance in thermogenic tissues. Given the primary role of BAT in thermoregulation, we assessed GPR84 expression in the BAT of mice (Figure 1E) and body temperature (Supplemental Figure 1C) at room temperature (RT) (23°C) and in cold conditions (6°C) over a 7-day period. Cold exposure resulted in a marked upregulation of GPR84 gene and protein expression, similar to the results observed for UCP1 (38), a specific BAT activation marker (Figure 1, E and F). Furthermore, we isolated the stromal vascular fraction (SVF) from BAT and induced its differentiation into brown adipocytes in vitro. Notably, GPR84 and UCP1 expression levels progressively increased during differentiation (Figure 1G and Supplemental Figure 1D), with GPR84 exhibiting plasma membrane expression in fully matured brown adipocytes (Supplemental Figure 1E). Collectively, these findings provide evidence for the enrichment of GPR84 within BAT and its potential regulatory role in brown adipocyte function.

### GPR84 deficiency leads to BAT dysfunction during aging.

**Figure 2:**
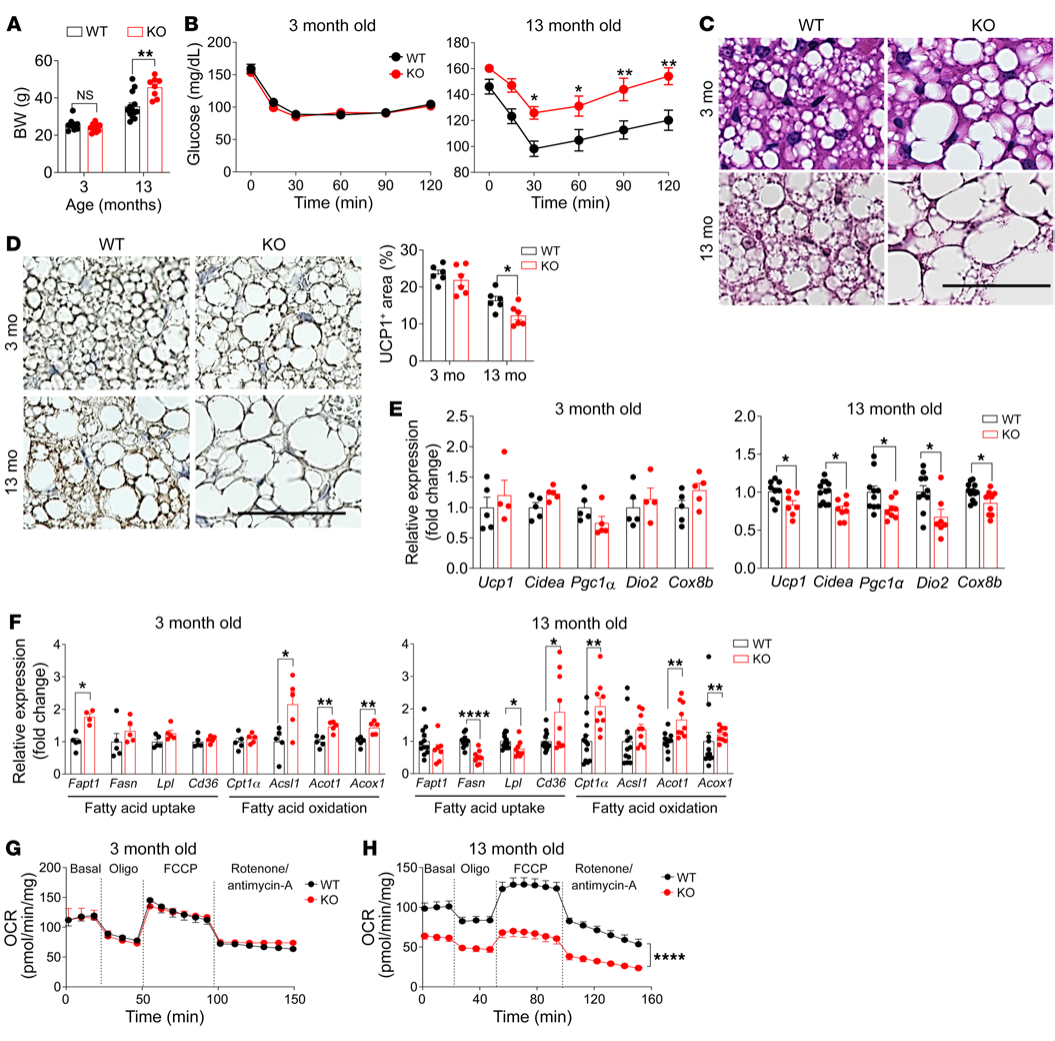
Phenotypes of GPR84-KO mice in young versus old mice. (**A**) Body weights (BW) of different ages of WT and GPR84-KO mice at RT. Data are represented as means ± SEM from at least 2 independent cohorts. *n* = 8–13/group/cohort. (**B**) Insulin tolerance test of WT and GPR84-KO mice on normal chow diet at different ages. *n* = 10/group. (**C**) H&E staining in BAT of WT and GPR84-KO mice at different ages at RT. Scale bar: 50 μm. (**D**) UCP1 staining and quantification of UCP1^+^ area in BAT of WT and GPR84-KO mice of different ages at RT. Scale bar: 50 μm. Data are representative images from at least 3 independent mouse cohorts. *n* = 6/group/cohort. (**E** and **F**) Thermogenic gene expression (**E**) and FA metabolism–related gene expression (**F**) were measured by qPCR in BAT from WT and GPR84-KO mice at different ages. *n* = 4–5/group for 3 months old; *n* = 9–10/group for 13 month old. (**G** and **H**) OCR was measured in BAT from WT and GPR84-KO mice at different ages. Data are represented as means ± SEM of at least 3 independent experiments in duplicate. *n* = 3–6/group. All data are represented as means ± SEM. *P < 0.05; **P < 0.01; ****P < 0.0001, 2-tailed Student’s *t* test (**A**, **D**, **E**, and **F**); 2-way ANOVA followed by Bonferroni’s multiple-comparison test (**B**, **G**, and **H**). See also Supplemental Figures 1 and 2.

To assess the function of GPR84, we first validated GPR84 expression in WT and whole-body GPR84-KO mice (Supplemental Figure 1, F and G) and then compared the phenotype of GPR84-KO mice and WT littermate control mice of different ages at RT. Aged (13 months old) GPR84-KO mice showed a marked increase in body weight (~10 g) compared with age-matched WT mice (Figure 2A and Supplemental Figure 2A), although food intake did not differ between the 2 groups. Body composition analysis revealed that aged KO mice stored more fat than age-matched WT mice (Supplemental Figure 2B). In addition, we found that aged GPR84-KO mice exhibited impaired insulin sensitivity compared with WT mice (Figure 2B), but no changes in glucose tolerance (Supplemental Figure 2C). Immunohistochemistry analysis showed increased WAT-like large unilocular lipid droplets with reduced UCP1 expression in the BAT of aged GPR84-KO mice compared with that in WT mice (Figure 2, C and D). Furthermore, we analyzed the expression of thermogenic genes in BAT of WT and KO mice at 3 months (young) and 13 months (aged). The expression of *Ucp1*, *CideA*, *Pgc1*α, *Dio2*, and *Cox8b* was downregulated in the BAT of aged KO mice compared with that in WT mice, but not in the BAT of young KO mice (Figure 2E). To confirm that changes in insulin sensitivity are associated with dyslipidemia in BAT (13, 39, 40), we analyzed genes related to FA metabolism in the BAT of WT and GPR84-KO mice (Figure 2F). The genes related to FA uptake were downregulated, except for *Cd36,* in aged GPR84-KO mouse BAT, whereas the genes related to FA oxidation (FAO) were markedly increased in both young and old GPR84-KO mouse BAT compared with age-matched WT mouse BAT (Figure 2F). We also examined the use of exogenous FAs in BAT of WT and GPR84-KO mice. As shown in Supplemental Figure 2D, GPR84-KO mice had a lower capability of using exogenous FAs than WT mice, suggesting impaired BAT function. Analysis of mitochondrial respiration in both young and aged BAT showed that the oxygen consumption rate (OCR) was not different between BAT from young WT and GPR84-KO mice (Figure 2G), whereas the OCR was approximately 50% lower in aged GPR84-KO mouse BAT than in aged WT mice (Figure 2H). Mitochondrial respiration in WAT did not differ between the 2 genotypes, regardless of age (Supplemental Figure 2, E and F). These results indicate that a lack of GPR84 in BAT accelerates age-mediated BAT dysfunction, which may lead to decreased systemic insulin sensitivity.

### GPR84 deficiency leads to mitochondria dysfunction in BAT upon cold exposure.

**Figure 3:**
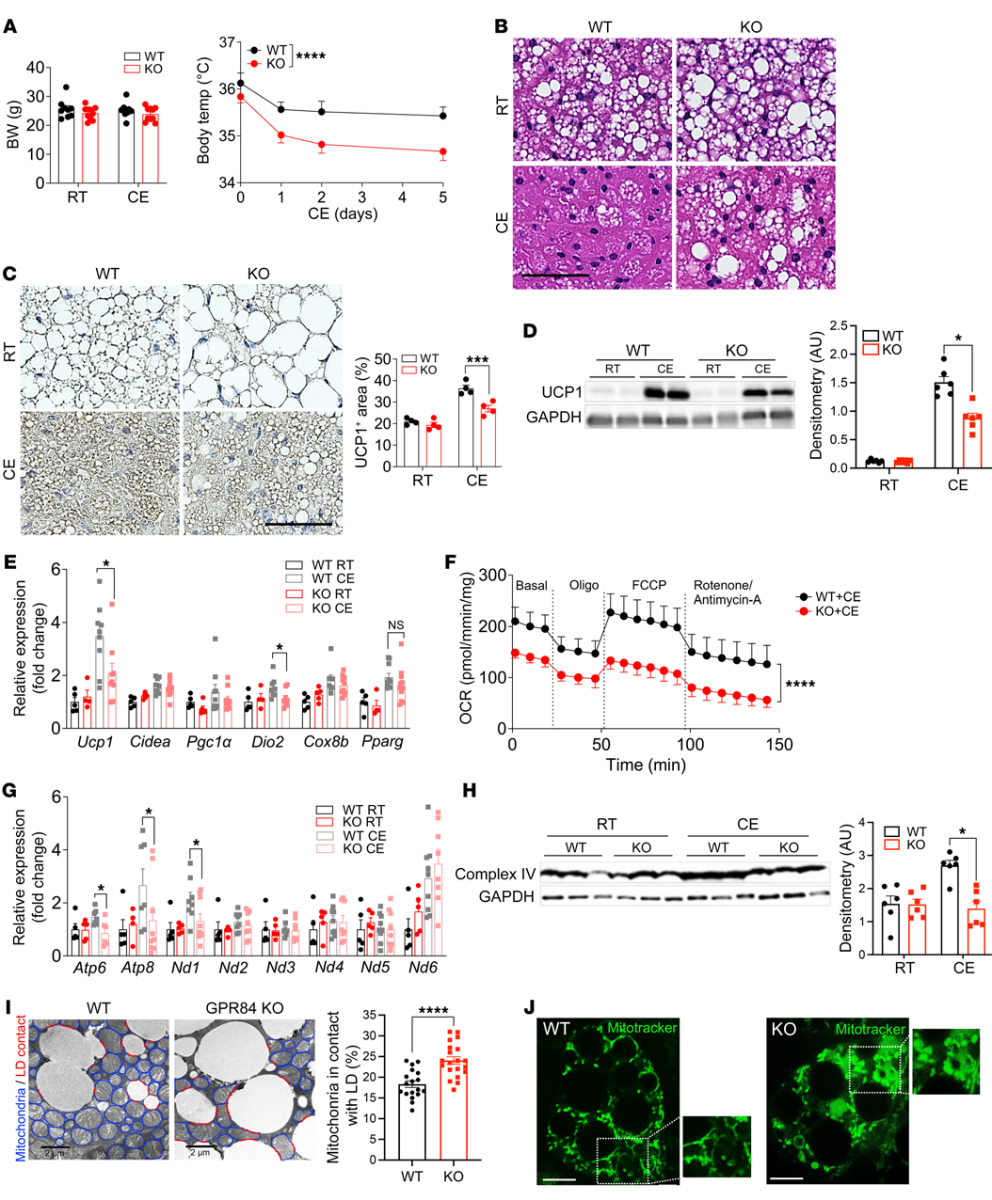
GPR84 deficiency leads to BAT dysfunction at cold exposure. (**A**) Body weight and body temperature of WT and GPR84-KO mice exposed to cold at 3 months of age. (**B** and **C**) Representative images of H&E (**B**) and UCP1 staining (**C**) in BAT from WT and GPR84-KO mice exposed to cold at 3 months of age. Scale bars: 50 μm. Images are representative of more than 5 images from at least 3 independent mouse cohorts. *n* =4/group/cohort. (**D**) Representative Western blotting analysis of UCP1 protein expression in BAT from WT and GPR84-KO mice at RT and with cold exposure. Image is representative of 3 independent experiments. Scanned bar graphs represent data as means ± SEM. *n* = 6/group. (**E**) Thermogenic gene expression levels were measured by qPCR in BAT of WT and GPR84-KO mice at RT and with cold exposure. Data are represented as means ± SEM of at least 3 independent experiments in triplicate. *n* = 5–9/group. (**F**) OCR was measured in BAT from WT and KO mice after 6 days of cold exposure. Data are represented as means ± SEM of at least 3 independent experiments in duplicate. *n* = 4/group. (**G**) Mitochondrial function–related gene expression levels were measured by qPCR in brown adipocytes isolated from WT and GPR84-KO mice at RT and with cold exposure. Data are represented as means ± SEM of at least 3 independent experiments in triplicate. *n* = 7–9/group. (**H**) Western blotting analysis of complex IV in BAT of WT and KO mice at RT and with cold exposure. *n* = 5/group. (**I**) TEM images of BAT from WT and KO mice 6 days after cold exposure. Images are representative of more than 20 images from at least 3 independent mouse cohorts. Scale bars: 2 μm. Mitochondria contacted with lipid droplets were quantified and plotted in the bar graph (right). *n* = 17–20/group. (**J**) Representative images of MitoTracker–green fluorescence for mitochondrial morphology were analyzed by confocal microscopy in brown adipocytes isolated from WT and KO mice. Scale bars: 50 μm. Insets are further magnified (×1.5) images of the selected area. *n* = 8–10/group. *P < 0.05; ***P < 0.001; ****P < 0.0001, 2-tailed Student’s *t* test (**C**, **D**, and **I**); 2-way ANOVA followed by Bonferroni’s multiple-comparison test (**A**, **E**, **F**, **G**, and **H**). See also Supplemental Figures 3 and 4.

To evaluate the role of GPR84 in BAT regardless of aging, we exposed young WT and GPR84-KO mice to cold (6°C) stimulation. No differences in body weight were found between the groups after 6 days of cold exposure (Figure 3A). Consistently, the weights of different metabolic organs, such as gonadal WAT (gWAT), BAT, and the liver, showed no changes after cold exposure (Supplemental Figure 3A). However, the body temperatures of GPR84-KO mice were significantly lower than those of WT mice (Figure 3A). Histological analysis revealed that BAT from KO mice had a more unilocular, WAT-like morphology, with reduced UCP1 levels, than BAT from WT mice 6 days after cold exposure (Figure 3, B–D). The mRNA expression levels of *Ucp1* and *Dio2* were also markedly lower in the BAT of KO mice than in WT mice after cold exposure (Figure 3E). In contrast, after cold exposure, the expression levels of WAT-selective genes were higher in BAT from KO mice than that from WT mice (Supplemental Figure 3B), which may explain the WAT-like morphology of BAT from the KO mice, even after cold exposure. Many recent studies have focused on the role of mitochondria in BAT, which promote energy consumption via adaptive thermogenesis (12, 15, 41–43). Thus, we measured the OCR to evaluate mitochondrial respiration and found that the OCR in KO mouse BAT was approximately 50% lower than that in WT mouse BAT 6 days after cold exposure (Figure 3F), whereas the OCR in other metabolic tissues, such as gWAT (Supplemental Figure 3C), inguinal WAT (iWAT) (Supplemental Figure 3D), and muscles (Supplemental Figure 3E), showed no differences in either genotype, indicating that GPR84-KO mice had decreased mitochondrial activation in BAT compared with WT mice after cold stimulation. However, we found that the number of mitochondria in BAT between WT and GPR84-KO mice did not differ after cold exposure by analyzing staining with Tim23, a component of mitochondrial inner-membrane import protein (Supplemental Figure 4A), and mitochondrial DNA as markers of mitochondrial biogenesis (Supplemental Figure 4B). Although mitochondrial numbers were not different between the 2 genotypes, mitochondrial function–related gene expression and complex IV (cytochrome *c* oxidase; last enzyme in the mitochondrial respiratory electron transport chain) protein levels after cold stimulation decreased in GPR84-KO mouse BAT compared with those in WT mouse BAT (Figure 3, G and H). Transmission electron microscopy (TEM) images to observe the morphology of mitochondria in BAT from WT and GPR84-KO mice showed that KO mouse BAT was packed with longer or bean-shaped mitochondria that contacted a much larger area of lipid droplets (peridroplet mitochondria) compared with WT mouse BAT packed with more round-shaped mitochondria, which are well-known cold-induced BAT mitochondrial morphologies (Figure 3I). Benador et al. reported that the contact area between mitochondria and lipid droplets decreases under cold exposure and that more peridroplet mitochondria in BAT lead to lipid droplet expansion and decreased FAO in BAT (44), which is the phenotype of GPR84-KO mice. Using green MitoTracker staining in WT and GPR84-KO brown adipocytes, mitochondria in WT cells appeared filamentous, rod-like in shape, whereas mitochondria in KO cells were swollen and spherical (Figure 3J).

Taken together, these findings demonstrate that lack of GPR84 decreases mitochondrial function and morphology in BAT, leading to an increased number of large lipid droplet–containing WAT-like adipocytes and dysfunctional BAT activation in young mice after cold exposure.

### GPR84 stimulation promotes brown adipocyte function.

**Figure 4:**
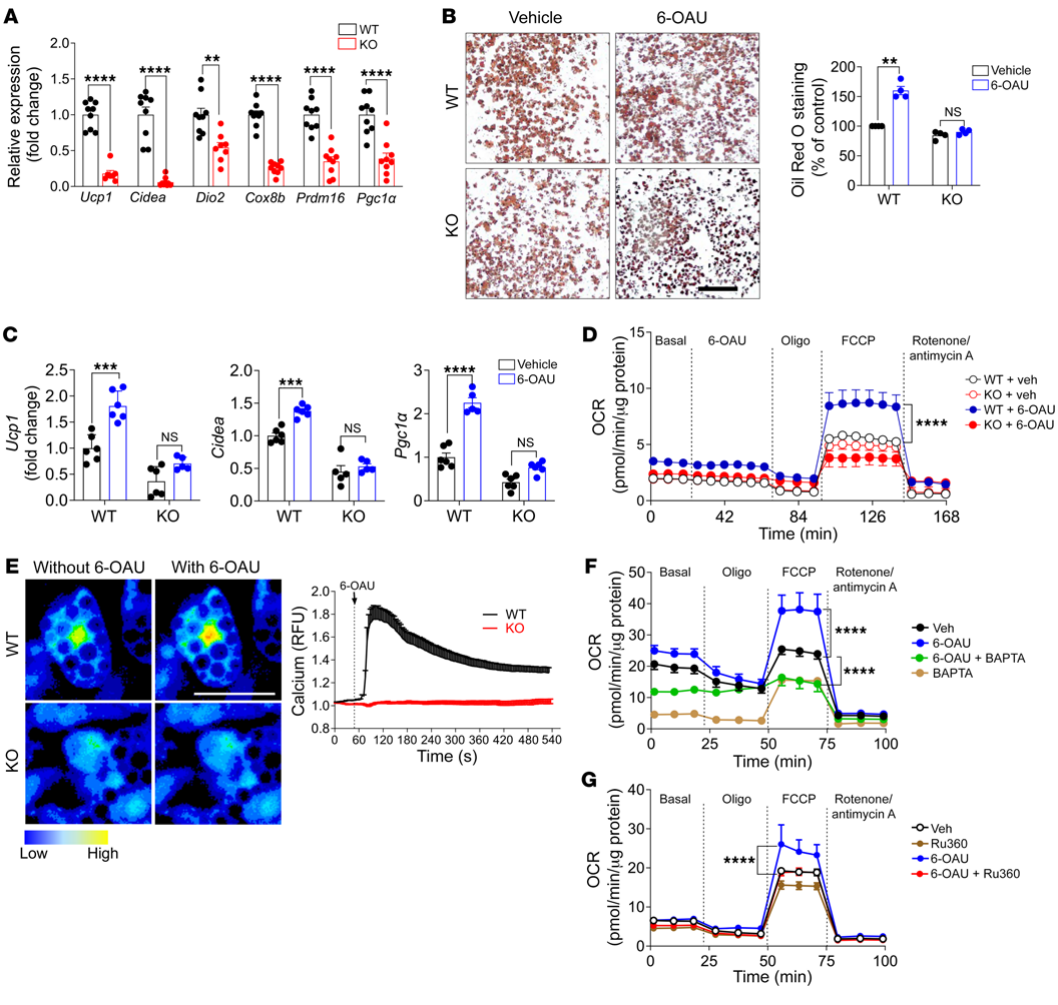
GPR84 stimulation promotes brown adipocyte function. (**A**) Thermogenic genes were measured by qPCR in fully differentiated brown adipocytes from WT and GPR84-KO mice. Data are represented as means ± SEM of at least 3 independent experiments. *n* = 9/group. (**B**) Images of Oil Red O staining in WT and KO brown adipocytes with or without GPR84 agonist 6-OAU treatment. Image is a representative image from 3 independent experiments. *n* = 5/group. Scale bar: 50 μm. (**C**) *Ucp1*, *Cidea*, and *Pgc1α* mRNA levels in WT and KO brown adipocytes with or without 6-OAU treatment. Data are represented as mean ± SEM of 2 independent experiments. *n* = 6/group. (**D**) OCR was measured in WT and KO brown adipocytes. Cells were pretreated with or without 6-OAU for 30 minutes before OCR was measured by a Seahorse X24 analyzer. Data are represented as means ± SEM of at least 3 independent experiments in duplicate. *n* = 5/group. (**E**) WT and GPR84-KO brown adipocytes were incubated with the calcium-sensitive dye Fluo-4 AM for 1 hour at RT, followed by live-cell imaging with a confocal laser-scanning microscope and stimulation with 6-OAU (50 μM). Data are representative images from more than 3 independent experiments. *n* = 6–10/group. Scale bar: 50 μm. (**F**) WT brown adipocytes were pretreated with 6-OAU (50 μM) for 1 hour, followed by treatment with BAPTA-AM for 30 minutes, and then OCR was measured. (**G**) Brown adipocytes were pretreated with 6-OAU (50 μM) for 1 hour and treated with Ru360 for 1 hour, and then OCR was measured. Data and images are representative of at least 3 independent experiments in duplicate. *n* = 5/group. **P < 0.01; ***P < 0.001; ****P < 0.0001, 2-tailed Student’s *t* test (**A**–**C**); 2-way ANOVA followed by Bonferroni’s multiple-comparison test (**D**, **F**, and **G**). See also Supplemental Figure 5.

Next, cultured primary brown adipocytes were used to evaluate whether GPR84 stimulation activates BAT in vitro. Thermogenic gene expression levels as well as adipocyte differentiation-related gene expression levels were significantly downregulated in GPR84-KO brown adipocytes compared with those in WT brown adipocytes (Figure 4A and Supplemental Figure 5A). Interestingly, adipocyte differentiation-related genes, such as *Ppar*γ, *Fabp4*, and adiponectin, were downregulated in isolated GPR84-KO brown adipocytes, but not in KO BAT, which contained increased numbers of WAT-like adipocytes (Supplemental Figure 5A). In contrast, treatment with the GPR84 agonist 6-*n*-octylaminouracil (6-OAU) (28) led to increased lipid droplet and thermogenic gene expression levels in mature brown adipocytes isolated from WT mice, whereas 6-OAU treatment had no effect on KO brown adipocytes compared with vehicle treatment (Figure 4, B and C). Furthermore, 6-OAU treatment increased mitochondrial respiration by approximately 50% in WT brown adipocytes, but not in KO brown adipocytes (Figure 4D). Moreover, any effects observed after GPR84 stimulation in BAT involved UCP1-mediated thermoregulation. Thus, we administered the β_3_-adrenergic receptor agonist CL-316243 to WT and GPR84-KO mice and found that KO mouse BAT and KO brown adipocytes had reduced responses to CL-316243 (which increased body temperature, mitochondrial respiration, and lipolysis) compared with WT mouse BAT and brown adipocytes, respectively (Supplemental Figure 4, C–E). These results clearly indicate that GPR84 regulates UCP1-mediated respiration in brown adipocytes. In addition, immunofluorescence results showed reduced UCP1 expression in brown adipocytes isolated from GPR84-KO mice compared with that in brown adipocytes of WT mice (Supplemental Figure 5B).

To explore the mechanism by which GPR84 stimulation affects brown adipocyte function, we first determined the GPR84 stimulation–mediated signaling pathway using a second messenger-specific luciferase reporter activity assay driven by a cAMP-responsive element (CRE-luc) for G_s_-coupled pathways and a serum-responsive element (SRE) for G_q/11_-coupled pathways. Since GPR84 is a known G_i/o_-coupled receptor that inhibits cAMP (28), HEK293 cells transiently expressing GPR84 were pretreated with forskolin to increase cAMP levels (45) and then treated with different concentrations of GPR84 agonists, including 6-OAU (28), medium-chain FAs (decanoic acid, C10 FA; lauric acid, C12 FA) (27), and embelin (46). The CRE-luc activity assay revealed that 6-OAU effectively inhibited forskolin-induced CRE-luc activity in a dose-dependent manner compared with other known GPR84 agonists (Supplemental Figure 5C). We verified that the 6-OAU–mediated inhibition of forskolin-induced CRE-luc activity was abolished in the presence of pertussis toxin (PTX), an inhibitor of G_i/o_ -coupled receptor activity (Supplemental Figure 5D). These data indicate that GPR84 primarily activates the PTX-sensitive G_i/o_ pathway to initiate downstream signaling. Activated G_i/o_ proteins release their Gβγ subunits, promoting calcium release from the ER (47). The G_i/o_-dependent pathway can affect intracellular Ca^2+^ responses. We found that 6-OAU treatment increased SRE-luc activity, which was stimulated by the G_q/11_ signaling pathway, as well as intracellular Ca^2+^ elevation (Supplemental Figure 5E). Since Ca^2+^ uptake and efflux through mitochondrial transporters and exchangers can affect mitochondrial function (48–50), we performed a Ca^2+^ mobility assay to explore whether GPR84 stimulation–mediated intracellular Ca^2+^ elevation affects mitochondrial function. Brown adipocytes isolated from WT and KO mice were labeled with the cell-permeable green fluorescent Ca^2+^ indicator Fluo-4 AM and then treated with 6-OAU to measure the intracellular Ca^2+^ release from the ER. As shown in Figure 4E, 6-OAU stimulation increased the intracellular Ca^2+^ concentration only in WT brown adipocytes, but not in KO cells (Figure 4E). Ca^2+^ elevation induced by 6-OAU stimulation in WT brown adipocytes was completely blocked by BAPTA-AM, an intracellular Ca^2+^ chelator, and 6-OAU had no effect on calcium release in WT cells (Supplemental Figure 5F), indicating that GPR84 stimulation induces intracellular Ca^2+^ release. Consequently, 6-OAU–induced mitochondrial respiration was blocked by BAPTA-AM (Figure 4F) and Ru360, a mitochondria-specific Ca^2+^ uptake blocker (51) (Figure 4G). These results indicate that GPR84 stimulation elevates intracellular Ca^2+^ concentration and promotes Ca^2+^ influx into the mitochondria to increase oxidative metabolism in brown adipocytes (Supplemental Figure 5G).

### GPR84 stimulation promotes BAT activation in vivo after cold exposure.

**Figure 5:**
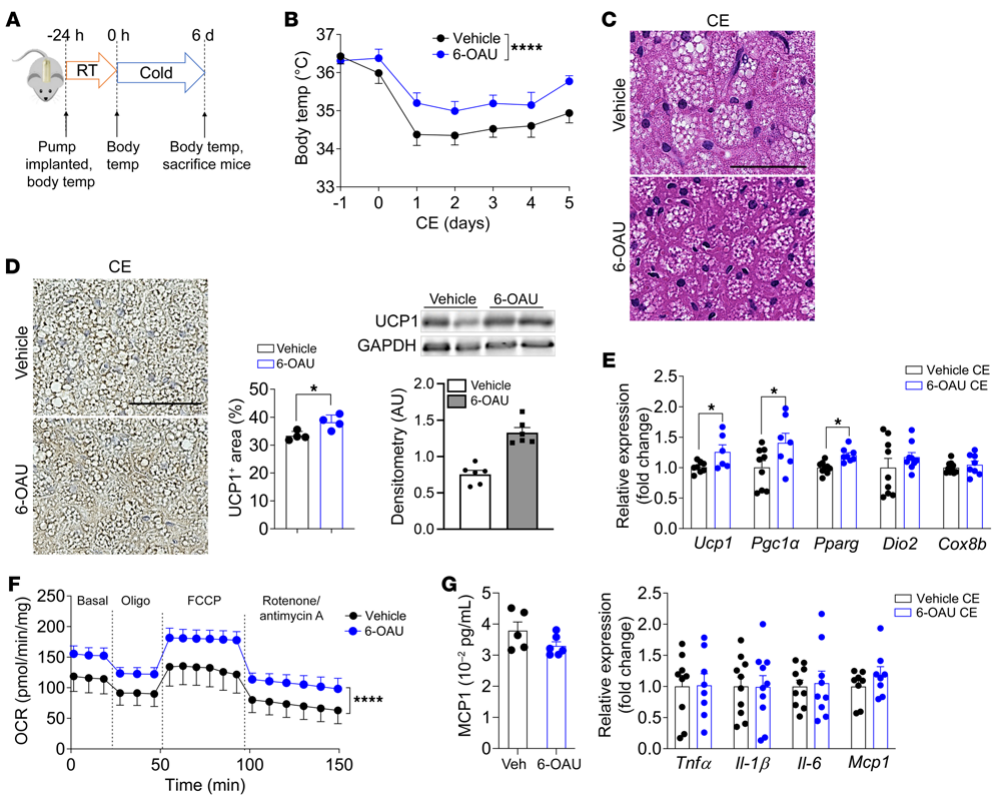
GPR84 agonist 6-OAU promotes BAT activation in mice at cold exposure. (**A**) Experimental design for 6-OAU in vivo treatment using osmotic minipump implantation. (**B**) Body temperature of vehicle- (veh) and 6-OAU–treated mice during cold exposure. *n* = 10/group. (**C**) H&E staining of BAT from vehicle- and 6-OAU–treated mice 6 days after cold exposure. Images are representative of more than 10 images from at least 3 independent mouse cohorts. *n* = 4/group/cohort. Scale bar: 50 μm. (**D**) UCP1 staining in BAT from vehicle- and 6-OAU–treated mice at 6 days after cold exposure. Images are representative of more than 10 images from at least 3 independent mouse cohorts. *n* = 4/group/cohort. Western blotting analysis of UCP1 protein expression in BAT from vehicle- and 6-OAU–treated mice at 6 days after cold exposure. The scanned bar graphs are expressed as the mean ± SEM. *n* = 6/group. Scale bar: 50 μm. (**E**) Thermogenic gene mRNA levels in BAT of vehicle- and 6-OAU–treated mice at 6 days after cold exposure. Data are represented as means ± SEM of at least 3 independent experiments in triplicate. *n* = 6– 8/group. (**F**) OCR of BAT from vehicle- and 6-OAU–treated mice at 6 days after cold exposure. Data are represented as means ± SEM of at least 3 independent experiments in duplicate. *n* = 4/group. (**G**) Plasma levels of MCP1 were measured by ELISA (*n* = 5–6/group) in vehicle- and 6-OAU–treated mice, and inflammatory gene expression was measured by qPCR (*n* = 5–10/group) in BAT from vehicle- and 6-OAU–infused mice at 6 days after cold exposure. *P < 0.05; ****P < 0.0001, 2-tailed Student’s *t* test (**D**, **E**, and **G**); 2-way ANOVA was followed by Bonferroni’s multiple-comparison test (**B** and **F**).

To evaluate the effect of GPR84 activation in vivo, WT mice were infused with 6-OAU using an osmotic minipump implanted under the skin in the interscapular region and then subjected to cold exposure (Figure 5A). Body and metabolic tissue weights were not altered during the 6 days of cold exposure. However, mice infused with 6-OAU maintained their core body temperature better than those infused with vehicle during cold exposure (Figure 5B). The BAT of 6-OAU–infused mice contained an increased number of multilocular brown adipocytes as well as an increased UCP1 protein level compared with that of vehicle-infused control mice (Figure 5, C and D). Consistently, thermogenic gene expression levels, including those of *Ucp1*, *PGC1α*, and *PPAR*γ*,* were upregulated in 6-OAU–infused mouse BAT (Figure 5E). Furthermore, the OCR in 6-OAU–infused mouse BAT was approximately 50% higher than that in the BAT of control mice (Figure 5F), whereas there were no changes in the OCR of iWAT and gWAT in either group (data not shown). Short-term injection of 6-OAU can increase inflammation in rats (28); thus, we determined whether the levels of inflammatory cytokines, such as TNF-α, IL-1β, IL-6, and MCP1, were changed by 6-OAU infusion. No differences were observed in plasma MCP-1 and inflammatory gene expression levels (Figure 5G), indicating that the effect of 6-OAU on BAT activation was independent of inflammation. Collectively, these findings revealed that GPR84 stimulation in vivo activates BAT function and regulates core body temperature during cold exposure.

### Brown adipocyte–specific GPR84 activation is required for thermogenesis.

**Figure 6:**
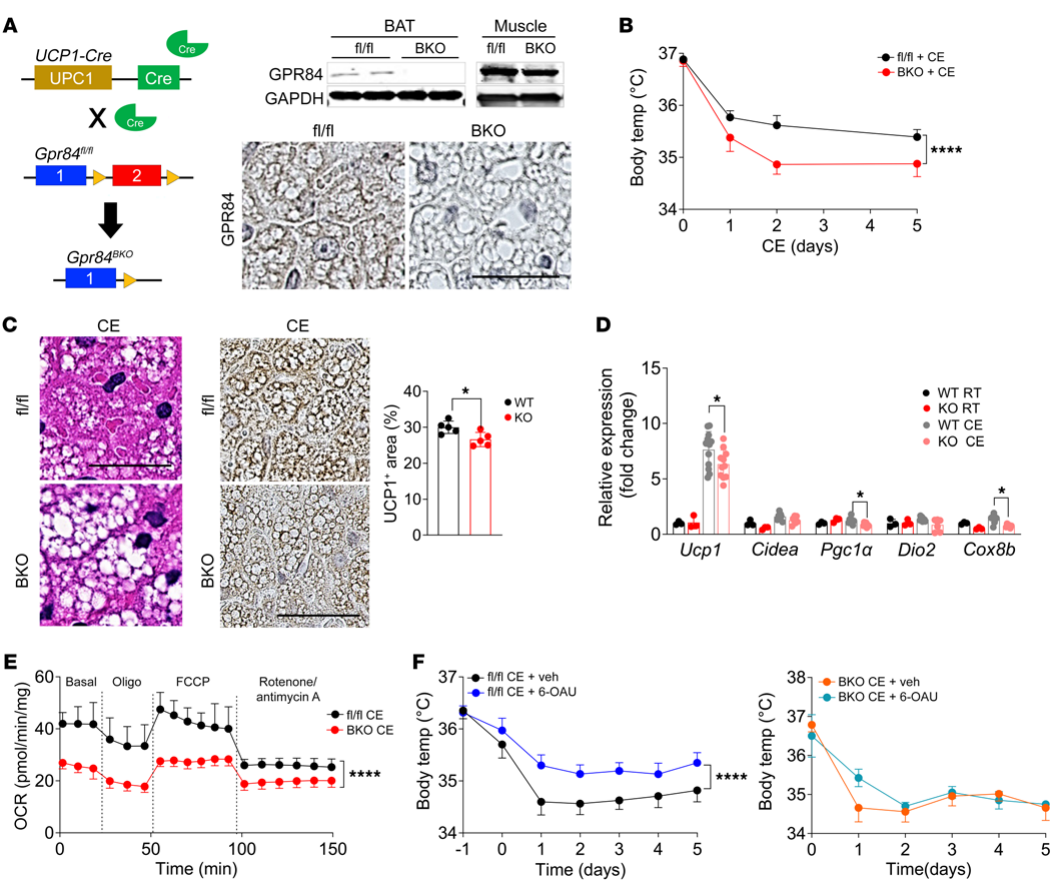
Brown adipocyte–specific GPR84 stimulation is required for thermogenesis. (**A**) Overview of the GPR84^BKO^ mouse model and validation of GPR84 protein expression in BAT from 2-month-old *Gpr84^fl/fl^* and GPR84^BKO^ mice. Western blotting analysis of GPR84 protein expression in brown adipocytes isolated from BAT and muscle of *Gpr84^fl/fl^* and GPR84^BKO^ mice. *n* = 3/group. Scale bar: 50 μm. (**B**) Body temperature changes of *Gpr84^fl/fl^* and GPR84^BKO^ mice exposed to cold at 3 months of age. *n* = 8/group. (**C**) H&E staining and UCP1 staining in BAT from *Gpr84^fl/fl^* and GPR84^BKO^ mice exposed to cold at 3 months of age. Images are representative of 6 images from 2 independent mouse cohorts. *n* = 5/group. Scale bars: 50 μm. (**D**) Thermogenic gene expression levels in BAT from *Gpr84^fl/fl^* and GPR84^BKO^ mice housed at RT or exposed to cold. Data are represented as means ± SEM for at least 3 independent experiments in triplicate. *n* = 3–10/group. (**E**) OCR of BAT from *Gpr84^fl/fl^* and GPR84^BKO^ mice at 6 days after cold stimulation. Data are represented as means ± SEM in duplicate. *n* = 4/group. (**F**) Body temperature changes of vehicle- and 6-OAU–treated *Gpr84^fl/fl^* and GPR84^BKO^ mice exposed to cold at 3 months of age. *n* = 4–15/group. *P < 0.05; ****P < 0.0001, 2-tailed Student’s *t* test (**C**); 2-way ANOVA followed by a Bonferroni’s multiple comparison test (**B**, **D**, **E**, and **F**). See also Supplemental Figure 6.

To characterize the specific role of GPR84 in BAT, we generated brown adipocyte–specific GPR84-KO mice (GPR84^BKO^) by crossing *Gpr84^fl/fl^* mice with *Ucp1-Cre* mice (Figure 6A). Immunohistochemistry and Western blotting revealed efficient deletion of GPR84 in brown adipocytes (Figure 6A). GPR84^BKO^ mice and control *Gpr84^fl/fl^* mice were exposed to cold in the same way as whole-body GPR84-KO mice, and we observed that the body temperatures of GPR84^BKO^ mice were significantly lower than those of control *Gpr84^fl/fl^* mice (Figure 6B) during cold exposure. GPR84^BKO^ mouse BAT exhibited a unilocular phenotype and decreased levels of UCP1 compared with *Gpr84^fl/fl^* mouse BAT (Figure 6C). Thermogenic gene expression levels were consistently lower in GPR84^BKO^ mouse BAT than in *Gpr84^fl/fl^* mouse BAT (Figure 6D). We then evaluated mitochondrial function and found that the OCR in GPR84^BKO^ mouse BAT was lower than that in *Gpr84^fl/fl^* mouse BAT at 6 days after cold exposure (Figure 6E), while we did not observe any statistical differences in the OCR of muscle (Supplemental Figure 6A), gWAT (Supplemental Figure 6B), and iWAT (Supplemental Figure 6C) between both genotypes, indicating that GPR84^BKO^ mice phenocopied GPR84 whole-body KO mice. Furthermore, we directly validated whether 6-OAU–mediated GPR84 stimulation had brown adipocyte–specific effects in vivo following 6-OAU infusion. GPR84^BKO^ mice and *Gpr84^fl/fl^* mice were infused with 6-OAU and then exposed to cold conditions. *Gpr84^fl/fl^* mice infused with 6-OAU maintained body temperature better than *Gpr84^fl/fl^* mice infused with the vehicle under cold conditions, whereas GPR84^BKO^ mice infused with 6-OAU showed no difference from those infused with vehicle (Figure 6F). Thermogenic gene levels increased upon 6-OAU infusion in *Gpr84^fl/fl^* mouse BAT, but not in GPR84^BKO^ mouse BAT (Supplemental Figure 6D). This is consistent with the phenotype observed in whole-body GPR84-KO mice. Taken together, these results indicate that BAT function regulated by GPR84 stimulation depends on brown adipocyte–specific GPR84 stimulation.

## Discussion

Targeting signaling pathways in adipose tissues is a promising approach to controlling their functions (2, 52, 53). GPR84 is a metabolic GPCR whose expression in leukocytes has been previously described (27). Although GPR84 is highly expressed in immune cells, its deficiency does not affect the inflammatory status in metabolic tissues such as skeletal muscle (43), liver (54), or adipose tissue, suggesting that GPR84 per se may not be involved in inflammatory regulation in these tissues. Furthermore, the potential role of GPR84 signaling in modulating metabolic responses has not yet been explored. In this study, we showed that GPR84 was highly expressed in human and mouse adipose tissues. In humans, the GPR84 expression levels in adipose tissue from obese and nondiabetic subjects were positively correlated with low-calorie diet intervention (Gene Expression Omnibus [GEO] GSE95640) (55) (Supplemental Figure 1A), suggesting a beneficial role for GPR84 in obesity. In mice, GPR84 is highly expressed in BAT, and its expression is increased by cold stimulation. These findings prompted us to investigate the role of GPR84 in the development of BAT. We demonstrated that GPR84 plays a crucial role in BAT activation under cold stimulation using GPR84 whole-body KO and brown adipocyte–specific GPR84-KO animal models, whereas animals with a lack of GPR84 showed cold intolerance and increased body weight during aging. Furthermore, GPR84 activation promotes brown adipocyte thermogenic activity and mitochondrial respiration through G_i/o_-dependent Ca^2+^ activation and improves overall BAT function following cold stimulation. Our data clearly indicate that GPR84 is a promising target for BAT stimulation for improving metabolic dysfunction.

Adipose tissue aging is a common feature of aging, and obesity plays a key role in the mechanisms and pathways of longevity, age-related diseases, inflammation, and metabolic dysfunction (56–58). Adipose tissue aging is associated with BAT whitening, WAT redistribution, and increased inflammation in adipose tissue (56–58). Various factors influence age-associated BAT involution, including mitochondrial dysfunction, sympathetic nervous system impairment, age-induced alterations in brown adipogenic stem/progenitor cell function, and changes in endocrine signaling (59). In this study, we demonstrated that GPR84-KO mice exhibited accelerated age-mediated body weight gain and BAT dysfunction compared with age-matched WT mice under ambient conditions (RT). Aged GPR84-KO mice (13 months old) showed impaired mitochondrial function (decreased OCR) and reduced expression of thermogenic genes in BAT, suggesting that GPR84 activation during aging attenuates the functional decline of BAT at RT. Furthermore, aged GPR84-KO mice showed decreased insulin sensitivity compared with age-matched WT mice (Figure 2B). The risks of developing type 2 diabetes mellitus and insulin resistance are associated with senile skeletal muscle dysfunction and other age-related factors. We assumed that aged GPR84-KO mice have peripheral insulin resistance; thus, a hyperinsulinemic-euglycemic clamp study to delineate the tissues responsible for decreased insulin sensitivity would be necessary. Although GPR84 is expressed in the skeletal muscle, GPR84-KO mice may have defective skeletal muscle function for insulin sensitization during aging. However, young mice (3 months old) exhibited no phenotypic differences between the 2 genotypes at RT.

Many studies have hypothesized that increased FAO is required for BAT thermogenesis (60). BAT contains dense mitochondria and requires FAs for heat production (61–63). Dysfunctional BAT has been implicated in the progression of lipid disorders, including increased glycolysis, FA synthesis, and FAO (64, 65). It is unclear how lipid storage and lipolysis in BAT balances to fuel heat generation. In this study, although aged GPR84-KO mice displayed increased FAO-related gene expression levels, mitochondria in KO BAT were dysfunctional. If increased FAO facilitates thermogenesis in aged GPR84-KO mice, thermogenesis would certainly increase in aged KO mouse BAT when FAO is maximal. However, contrary to this hypothesis, the thermogenic function of aged KO BAT was decreased (Figure 2). Interestingly, we found that aged KO mice had problems utilizing exogenous FAs (Supplemental Figure 2D), which proves that aged KO mouse BAT has lower mitochondrial capability and higher demands for FAs compared with WT mouse BAT. The increased expression of FAO-related genes in BAT of KO mice may be due to a compensatory mechanism for overcoming mitochondrial dysfunction. Thus, this result supports our conclusion that the BAT of KO mice has a dysfunctional phenotype.

Adaptive thermogenesis has attracted attention because of its ability to increase systemic energy expenditure and counter obesity and diabetes (6, 66, 67). BAT is essential for classical nonshivering thermogenesis and cold acclimation–recruited norepinephrine-induced thermogenesis. β_3_-Adrenergic receptor signaling is the dominant signaling pathway controlling nonshivering thermogenesis in brown and beige adipocytes (68). Upon cold exposure, norepinephrine released from the sympathetic nervous system mainly binds to the β_3_-adrenergic receptor to induce adrenergic signaling on lipid droplets to promote lipolysis (69, 70). FAs produced upon lipolysis can undergo β oxidation to eventually produce NADH and flavin adenine dinucleotide (FADH_2_) for use in the electron transport chain during UCP1-mediated thermogenesis (71). Thus, to explore the biological effects of GPR84 stimulation in BAT, it was important to ensure that any effects observed upon GPR84 stimulation in BAT involved UCP1-mediated thermoregulation. As shown in Supplemental Figure 4, C–E, GPR84-KO mouse BAT and brown adipocytes had reduced responses to CL-316243 compared with WT mouse BAT and brown adipocytes, indicating that GPR84 stimulation regulates UCP1-dependent respiration in brown adipocytes.

New thermogenic pathways have been discovered in BAT in recent years. Ikeda et al. identified a noncanonical thermogenic mechanism by which beige fat controls whole-body energy homeostasis via Ca^2+^ cycling (72, 73). GPR84 signaling through a G_i/o_-coupled pathway activates Ca^2+^ release from the ER (47). Our results revealed that GPR84 stimulation induced intracellular Ca^2+^ release, which promoted mitochondrial respiration in primary brown adipocytes (Figure 4, E–G). Mitochondrial morphology, depolarization, fission, and fusion are coupled with Ca^2+^ mobilization (74, 75). However, the precise mechanism by which GPR84 stimulation–mediated Ca^2+^ mobilization controls mitochondrial function and BAT thermogenesis requires further in-depth study.

The mitochondria are dynamic cellular organelles. Our TEM data showed that WT BAT was packed with more round-shaped mitochondria, which is a well-known cold-induced BAT mitochondrial morphology, whereas GPR84-KO mouse BAT was packed with longer or bean-shaped mitochondria that contacted a much larger area of lipid droplets (peridroplet mitochondria) (Figure 3I). The high level of mitochondria–lipid droplet contact has been demonstrated in several studies, which showed that mitochondria–lipid droplet interaction enhances lipid droplet expansion rather than oxidation in BAT (41, 44, 76), which may be a reason for the larger lipid droplets in GPR84-KO mouse BAT than in WT mouse BAT. As mitochondrial staining in GPR84-KO brown adipocytes showed mitochondria that were swollen and spherical compared with those in WT cells in Figure 3J, mitochondrial swelling is a hallmark of mitochondrial dysfunction and is involved in the pathogenesis of many human diseases associated with oxidative stress, such as neurodegenerative diseases, cardiac ischemia, hypoxia, inflammation, and diabetes (77–79), which can indicate possible mitochondrial dysfunction in GPR84-KO brown adipocytes. Correlative functional studies of GPR84 stimulation as well as GPR84 loss of function are required so that we can better understand the relationship between the observed differences in mitochondrial structure shown with TEM and confocal microscopy analysis, particularly the role of mitochondrial transition permeability, Ca^2+^ regulation, and ATP levels during cold exposure.

Recent studies have shown that BAT activation is an effective approach in cancer therapy (80) and benefits patients with Alzheimer’s disease (AD) (81–83). Cold-stimulated BAT mitigates glucose uptake in the tumor tissues and is used for BAT-mediated thermogenesis at cold temperatures (80). As humans age, the ability of an individual’s organ to maintain its physiological temperature declines, as was observed in an aged mouse study. These changes in BAT function during aging may explain thermoregulatory deficits in the elderly and the underlying mechanisms of AD (82). Our GPR84 agonist 6-OAU infusion study showed that GPR84 activation improved BAT function in vivo without increasing inflammation (Figure 5G), although a potential role for GPR84 in the regulation of inflammation has been suggested by several groups (27, 28, 30). Therefore, GPR84 stimulation–mediated mechanisms of action in BAT for cancer metabolism and AD could be an interesting topic for future studies.

## Methods

### Animal care and use.

GPR84-KO mice were purchased from DeltaGene and housed at a specific pathogen–free facility (University of Texas Southwestern Medical Center). Male C57BL/6 (WT) or GPR84-KO littermates, from 8 weeks of age, were fed a normal chow (13.5% fat; LabDiet) or high-fat diet (60% fat; catalog D12492; Research Diet) ad libitum for 14 weeks. Mice received a fresh diet weekly, and food consumption and body weight were monitored. Twelve-week-old mice were housed at 6°C for 6 days. During cold exposure, the mouse body temperature was monitored every 1 or 2 days.

### Plasma triglycerides measurement.

After mouse euthanization, analysis of plasma total cholesterol, triglyceride, and FFA was performed on whole-blood samples obtained by venipuncture and centrifuged at 3,000*g* for 15 minutes. Samples were analyzed using a Vitros 250 Chemistry System (Ortho Clinical Diagnostics).

### Calcium mobility assay.

Differentiated brown adipocytes were incubated with the calcium-sensitive dye Fluo-4 AM for 15 minutes at 37°C and another 15 minutes at RT in DMEM without FBS (11). Cells were ready for the assay after being washed twice with a live-cell image solution. Using a confocal laser scanning microscope, images were taken every 4 seconds in the presence or absence of 6-OAU in both WT and GPR84-KO cells. Images and videos were recorded and analyzed using OpenLab imaging software, version 3 (Improvision Inc.).

### RNA isolation, cDNA synthesis, and qPCR.

Total RNA was extracted from BAT and primary brown adipocytes using an RNA Purification Kit (QIAGEN). cDNA was synthesized using SuperScript III and random primers. Quantitative PCR (qPCR) was performed in 10 μL reactions with SYBR Green Master Mix (Applied Biosystems) using an ABI Real-Time PCR System (Applied Biosystems). Relative gene expression was normalized to that of the standard housekeeping gene (RPL19) using the ΔΔCT method. The specificity of the PCR amplification was verified by melting curve analysis of the final products using the ABI 7500 system, version 2.3. The primer sequences are available in Supplemental Table 1.

### Cell culture.

Brown preadipocytes were cultured in DMEM (Corning) supplemented with 10% FBS, penicillin-streptomycin, and gentamicin (no. 15750060, Gibco, Thermo Fisher Scientific) in a humid incubator with 10% CO_2_ at 37°C. Preadipocytes were differentiated with an induction medium including 0.5 mM 3-isobutyl-1-methylxanthine (IBMX), 1 μM dexamethasone, and 5 μg/mL insulin to initiate in vitro differentiation for 2 days after the cells reached over 95% confluence. After 48 hours of incubation, cells were cultured in maintenance medium containing only 5 μg/mL insulin for 6 days.

### Tissue histology and immunohistochemistry.

Mouse BAT was paraffin embedded, cut in 4 μm sections, and stained with H&E, GPR84, and UCP1 (performed by the University of Texas Southwestern Medical Center Histology Core). Images (×100 or ×200 magnification) were acquired using the FSX100 Inverted Microscope (Olympus), and representative histological images are shown. Paraffin-embedded slides were prepared by the University of Texas Southwestern Medical Center Histology Core for immunohistochemistry staining. Briefly, deparaffinized sections were stained with anti-GPR84 (1:50) and anti-UCP1 (1:500) primary antibodies and incubated overnight at 4°C. Then the secondary antibodies (Life Technologies) were added for 2 hours, and finally, the coverslips were added.

### Brown adipocyte immunofluorescence staining.

Fully differentiated brown adipocytes were fixed for 10 minutes at RT and washed with PBS. The cells were then blocked and permeabilized with 10% normal goat serum and 0.5% Triton X-100 in PBS for 1 hour. Cells were incubated at 4°C overnight with the following primary antibody: anti-GPR84 (1:50, Bioss, bs-13507R-TR). Next, the cells were incubated with secondary antibodies and BODIPY (1:10000) at RT. Images were acquired using a confocal microscope (Zeiss LSM 880 with Airyscan) and analyzed using ImageJ (NIH).

### Cellular oxygen-consumption measurements.

Mitochondrial respiration was examined using a Seahorse XF24 Extracellular Flux Analyzer (Agilent) according to the manufacturer’s instructions. Briefly, mitochondrial respiration was determined following the manufacturer-recommended basal-oligomycin-FCCP-antimycin A/rotenone (BOFA) protocol 17. Ex vivo and in vitro mitochondrial function were measured using 3 to 5 mg BAT- and SVF-differentiated brown adipocytes, respectively. For tissues and cultured cells, 6-OAU (50 μM), oligomycin (2 μM), FCCP (8 μM), and antimycin A (10 μM) plus rotenone (3 μM) were injected. OCR was acquired using the Seahorse instrument.

### FAO measurement.

FAO was determined by the Agilent Seahorse XF palmitate oxidation stress test. Ex vivo FAO was measured using 3 to 5 mg BAT. After incubating the FAO medium for 1 hour, palmitate covalently conjugated to BSA and DMSO mixed with BSA (vehicle control) were added to initiate the XF assay. The following pathway agents were injected: 6-OAU (50 μM), oligomycin (2 μM), FCCP (8 μM), and antimycin A (10 μM) plus rotenone (3 μM). OCR was acquired using the Seahorse instrument.

### TEM.

Briefly, BAT from different mice was fixed by perfusion with a fixation buffer (0.1 mM sodium cacodylate containing 4% paraformaldehyde and 1% glutaraldehyde). The harvested tissue was then transferred to 2.5% glutaraldehyde in 0.1 mM sodium cacodylate buffer and cut into pieces. Next, the tissue pieces were sent to the University of Texas Southwestern Medical Center Electron Microscopy Core for subsequent sectioning and imaging processing. TEM photos were acquired using a JEOL 1200EX transmission electron microscope (JEOL).

### Oil Red O staining.

Differentiated brown adipocytes were fixed with 4% paraformaldehyde (15 minutes, RT) and rinsed 3 times with PBS. Cells were stained with 0.15% Oil Red O solution (60 minutes, RT), after which they were washed with ddH_2_O 4 times. The cells were then ready for photographs.

### Body-composition analysis.

The body weight of mice was measured with a scale, and total body lean and fat mass were measured with the Bruker Minispec mq10 System (Bruker).

### 6-OAU infusion in mice and body temperature monitoring.

Minipumps (1007D; Alzet) were implanted subcutaneously in 11- to 12-week-old mice to deliver 6-OAU (6 mg/kg/day) or vehicle (PBS). For body-temperature measurements, mice were infused with 6-OAU or vehicle for 2 weeks, and body temperature was measured using an Analysis System (Visitech Systems). Minipumps were implanted 7 days later for infusion of 6-OAU or vehicle.

### Antibodies and reagents.

Anti-GPR84 antibody (bs-13507R-TR) for immunofluorescence staining was purchased from Bioss Antibodies. Anti-UCP1 antibody (ab10983) was purchased from Abcam. Anti-TIM23 (sc-514463) was obtained from Santa Cruz Biotechnology Inc. 6-OAU (GPTL5846) was obtained from MedChemExpress. The Fluo-4 Calcium Imaging Kit (F10489) was obtained from Thermo Fisher Scientific. Unless specifically indicated, all other reagents were obtained from MilliporeSigma. More resources are listed in Supplemental Table 2.

### Statistics.

Data are presented as means ± SEM. The significance of differences between the groups was evaluated using ANOVA. Data were judged to be statistically significant at *P* < 0.05 by 2-tailed Student’s *t* test or 2-way ANOVA followed by Bonferroni’s post hoc test, where appropriate. Statistical parameters, including the exact value of *n*, the definition of center, dispersion, and precision measures (mean ± SEM), and statistical significance, are reported in figures and figure legends. Statistical analysis was performed using GraphPad Prism 9.

### Study approval.

all animal procedures were approved by the IACUC of The University of Texas Southwestern Medical Center (APN no. 2016-101841-G).

### Data availability.

RNA-Seq data sets for human samples were obtained from the GTEx database. Values for all data points in graphs are reported in the Supporting Data Values file.

## Author contributions

XNS and DYO conceived and designed the study. XNS performed most of the experiments, with help from YAA and MYW (Seahorse experiments); VAP, CODS, LMAB, ASC, JANC, SC, and MEI (mouse surgeries, tissue collection, and sample preparation); CL (Western blotting); HAK and WL (calcium mobility assay); and LV and RKG (primary adipocyte culture). RKG provided the key reagent. DYO and XNS analyzed and interpreted data and cowrote the manuscript.

## Supplementary Material

Supplemental data

Supporting data values

## Version 1 10/19/2023

In-Press Preview

## Version 2 12/15/2023

Electronic publication
