# Supplementary material for: GPR84-mediated signal transduction affects metabolic function by promoting brown adipocyte activity
Source: J Clin Invest. 2023 Dec 15;133(24):e168992. doi: 10.1172/JCI168992 (PMC10721148; doi:10.1172/JCI168992)
Supplement: Supplemental data [file jci-133-168992-s195.pdf]

SUPPLEMENTAL INFORMATION

GPR84-mediated signal transduction affects metabolic function by promoting brown adipocyte activity

Xue-Nan Sun[1], Yu A. An[1,2], Vivian A. Paschoal[1], Camila O. de Souza[1], May-yun Wang[1], Lavanya Vishvanath[1,3], Lorena M. A. Bueno[1], Ayanna S. Cobb[1], Joseph A. Nieto Carrion[1], Madison E. Ibe[1], Chao Li[1], Harrison A. Kidd[4], Shiuhwei Chen[1], Wenhong Li[4], Rana K. Gupta[1,3], and Da Young Oh[1,*]

[1]Touchstone Diabetes Center, Department of Internal Medicine, University of Texas Southwestern Medical Center, Dallas, TX 75390, USA, [2]Department of Anesthesiology, McGovern Medical School, The University of Texas Health Science Center, Houston, TX 77225, USA, [3]Division of Endocrinology, Department of Medicine, Duke Molecular Physiology Institute, Durham, NC 27701, USA, [4]Department of Cell Biology, University of Texas Southwestern Medical Center, Dallas, TX 75390, USA

* To whom correspondence should be addressed; Da Young Oh, PhD

E-mail: dayoung.oh@utsouthwestern.edu

Supplemental Table 1. Primers used for qPCR

Gene Symbol	Primer forward (5' → 3')	Primer reverse (5' → 3')
Gpr84	TCCAATTCTGTCTCCATCCT	CTGACTGGCTCAGATGAAA
Ucp1	AAGCTGTGCGATGTCCATGT	AAGCCACAAACCCTTTGAAAA
Dio2	TTGGGGTAGGGAATGTTGGC	TCCGTTTCCTCTTTCCGGTG
Cidea	TCCTATGCTGCACAGATGACG	TGCTCTTCTGTATCGCCCAGT
Pgc1α	TCCTCACACCAAACCCACAGAA	TTGGCTTGAGCATGTTGCGA
Pparg	GCCCTTTGGTGACTTTATGGA	GCAGCAGGTTGTCTTGGATG
Cox8b	TGCTGGAACCATGAAGCCAAC	AGCCAGCCAAAACTCCCACTT
Prdm16	ACACGCCAGTTCTCCAACCTGT	TGCTTGTTGAGGGAGGAGGTA
Resistin	AAGAACCTTTCATTTCCCCTCCT	GTCCAGCAATTTAAGCCAATGTT
Leptin	ATGTGCCCTTCCGATATACAACC	CGTGTCATCCACTAATCTTCTGG
Tie3	GAGACTGAACACAATCCTAGCC	GGAGTCCACGTACCCCGAT
Gsta3	AGATCGACGGGATGAAACTGG	CAGATCCGCCACTCCTTCT
Zfp423	CAGGCCCACAAGAAGAACAAG	GTATCCTCGCAGTAGTCGCACA
Mcp1	TTAAAAACCTGGATCGGAACCAA	GCATTAGCTTCAGATTTACGGGT
Il6	CCAGAGATACAAAGAAATGATGG	ACTCCAGAAGACCAGAGGAAAT
Tnfα	GCCACCACGCTCTTCTGCCT	GGCTGATGGTGTGGGTGAGG
IL-1β	AAATACCTGTGCCCTTGGGC	CTTGGCATCCACACTCTCCAG
Cycs	CCAGGTATACAAGCAGGTGTGCTC	CATCATTAGGGCCATCCTGGAC
Cox2	AGTTGATAACCGAGTCGTTCTGCCA	TCGGCCTGGGATGGCATCAGT
Cox4	ATTGGCAAGAGAGCCATTTCTAC	CACGCCGATCAGCGTAAGT
Cox6c	GGGAAGGACGTTGGTGTAGA	CTTATAGGCAGCGGCAACTC
Cox8a	TTCCTGCTTCGTGTGTTGTC	GATTGCAGAAGAGGTGACTGG
Nd1	CAGCCGGCCCATTCGCGTTA	AGCGGAAGCGTGGATAGGATGC
Nd2	TCCTCCTGGCCATCGTACTCAACT	AGAAGTGGAATGGGGCGAGGC
Nd3	ACCCTACAAGCTCTGCACGCC	GCTCATGGTAGTGGAAGTAGAAGGGCA
Nd4	TCGCCTACTCCTCAGTTAGCCACA	TGATGATGTGAGGCCATGTGCGA
Nd5	TCGGAAGCCTCGCCCTCACA	AGTAGGGCTCAGGCGTTGGTGT
Nd6	AATACCCGCAAACAAAGATCACCCAG	TGTTGGGGTTATGTTAGAGGGAGGGA
Atp6	GCTCACTCGCCCACTTCCTTCC	GCCGGACTGCTAATGCCATTGGTT
Atp8	ATGCCACAACTAGATACATCAACA	GGGGTAATGAATGAGGCAAA
Rpl19	ATGAGTATGCTCAGGCTACAGA	GCATTGGCGATTTCATTGGTC

Supplemental Table S2. Resources table

REAGENT or RESOURCE	SOURCE	IDENTIFIER
ANTIBODIES		
GPR84 polyclonal antibody	Bioss	Cat# bs-13507R-TR
Anti-UCP1 antibody	Abcam	Cat# ab10983
Anti-Tim23 antibody (H-8)	Santa Cruz	Cat# sc-514463
PLASMIDS		
SRE-Luciferase	Oh et al., 2010	N/A
CRE-Luciferase	Oh et al., 2010	N/A
GPR84 Plasmid	Origene	Cat#: MR206206
pcDNA3	Oh et al., 2010	N/A
CHEMICALS, PEPTIDES, AND RECOMBINANT PROTEINS		
One Shot® MAX Efficiency® Competent Cells	Invitrogen	Cat#12297-016
SuperFect Transfection Reagent	QIAGEN	Cat# 301305
EndoFree® Plasmid Purification Maxi Kit	QIAGEN	Cat# 12362
Oil RedO	Sigma-Aldrich	Cat# 0625-25G
Collagenase B	Roche	Cat# 11088815001
Normal Goat Serum	Vector Laboratories	Cat# S-1000
HEPES	Sigma-Aldrich	Cat# 83264
HBSS	Gibco	Cat# 14065-056
BSA fraction V	Sigma-Aldrich	Cat# A7888
QIAzol Lysis Reagent	Qiagen	Cat# 79306
Random Primers	Promega	Cat# C1181
M-MLV Reverse Transcriptase	Promega	Cat# M1701
RNasin® Ribonuclease Inhibitor	Promega	Cat# N2511
PowerUp™ SYBR™ Green Master Mix	Applied Biosystems	Cat# A25742
Embelin	Sigma-Aldrich	Cat# E1406-10MG
Lauric acid	Sigma-Aldrich	Cat# 143-07-7
Capric acid	Sigma-Aldrich	Cat# C1875-100G
6-OAU	MedChemExpress	Cat# HY-12764
CRITICAL COMMERCIAL ASSAYS		
**RNA purification kit**	**Qiagen**	**Cat# 74104**

Dual luciferase reporter assay system	Promega	Cat# E1910
Ultra Sensitive Mouse Insulin ELISA	Crystal Chem	Cat# 90082
Pierce™ BCA Protein Assay Kit	Thermo Scientific	Cat# 23225
Recombinant Mouse CCL2/JE/MCP-1	R&D	Cat# 479-JE-050/CF
DAB QUANTO	Thermo Fisher	Cat# TA-060-QHDX
Fluo-4 Calcium imaging kit	Thermo Fisher	Cat# F10489
EXPERIMENTAL MODELS: CELL LINES		
HEK293 cell	ATCC	Cat# CRL-1573
Primary brown adipocytes	This paper	N/A
EXPERIMENTAL MODELS: ORGANISMS/STRAINS		
Mouse: WT C57BL6/J	Jackson Labs	JAX:000664
Mouse: GPR84 KO mice	Deltagen (Lexicon)	
OLIGONUCLEOTIDES		
Primers, see Table S1	This paper	N/A
Software and Algorithms		
ImageJ	NIH	RRID: SCR_003070
Prism8	Graphpad	RRID:SCR_002798

Supplemental References

1. Tran KV, et al. Human thermogenic adipocyte regulation by the long noncoding RNA LINC00473. *Nat Metab.* 2020;2(5):397-412.

Supplemental Figure 1. Sun *et al*. ---- related to Figures 1 and 2

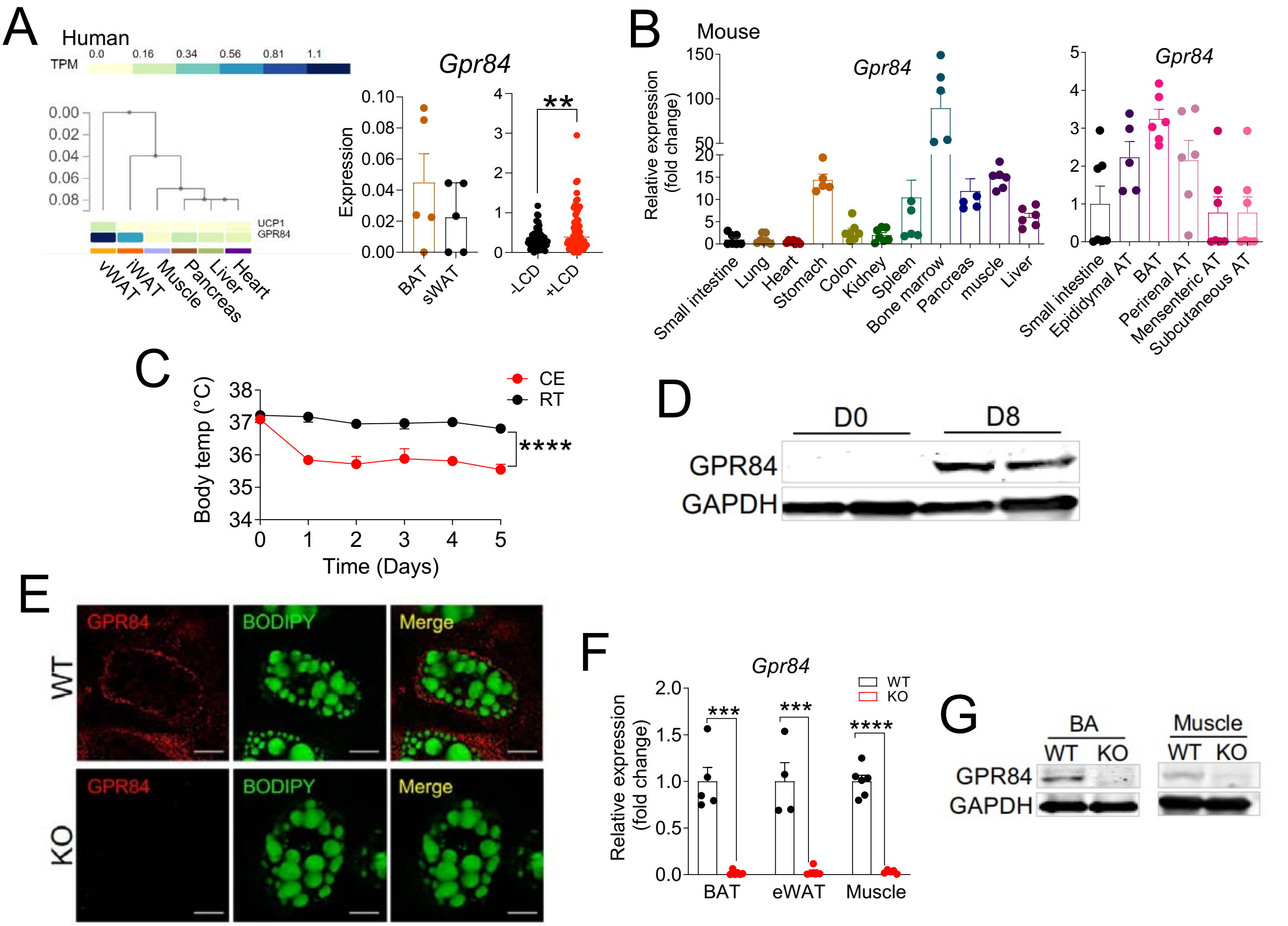

Figure S1- Related to Figures 1 and 2. Validation of GPR84 KO mice and GPR84 expression in different tissues.
(A) Expression of GPR84 correlated with the expression of UCP1. Data from the GTEx database. TPM normalized expression values of human BAT and sWAT (bar graph). Data from GSE150119 (1). TPM normalized GPR84 expression level of obese human sWAT before and after low calorie diet (- LCD vs. + LCD; scatter plot graph). Data from GSE95640. **(B)** mRNA expression pattern of GPR84 shown in different tissues or organs of mouse. n = 5 – 7/sample. **(C)** Body temperature of WT mice housed at RT and exposed to cold. n = 9/group. **(D)** Western blotting analysis of GPR84 protein expression in immature (D0) and mature (D8) brown adipocytes isolated from WT mice. Image is a representative image from three independent experiments. n = 5/group. **(E)** Representative immunofluorescence staining of GPR84 (red) and lipid droplets (BODIPY, green) in mature brown adipocytes isolated from WT and KO mice. Images are representative images from more than 10 cells of each three independent experiments. Scale bar = 10 µm **(F)** GPR84 mRNA expression in various tissues from WT and GPR84 KO mice. n = 6/group. **(G)** Western blotting analysis of GPR84 protein expression in mature brown adipocytes (BA) isolated from BAT and muscle of WT and GPR84 KO mice. ****, P < 0.0001; ***, P < 0.001; *, P < 0.05 by two-tailed Student's *t*-test (**A**, **F**); two-way ANOVA followed by a Bonferroni's multiple comparison test (**C**).

Supplemental Figure 2. Sun *et al*. ---- related to Figure 2

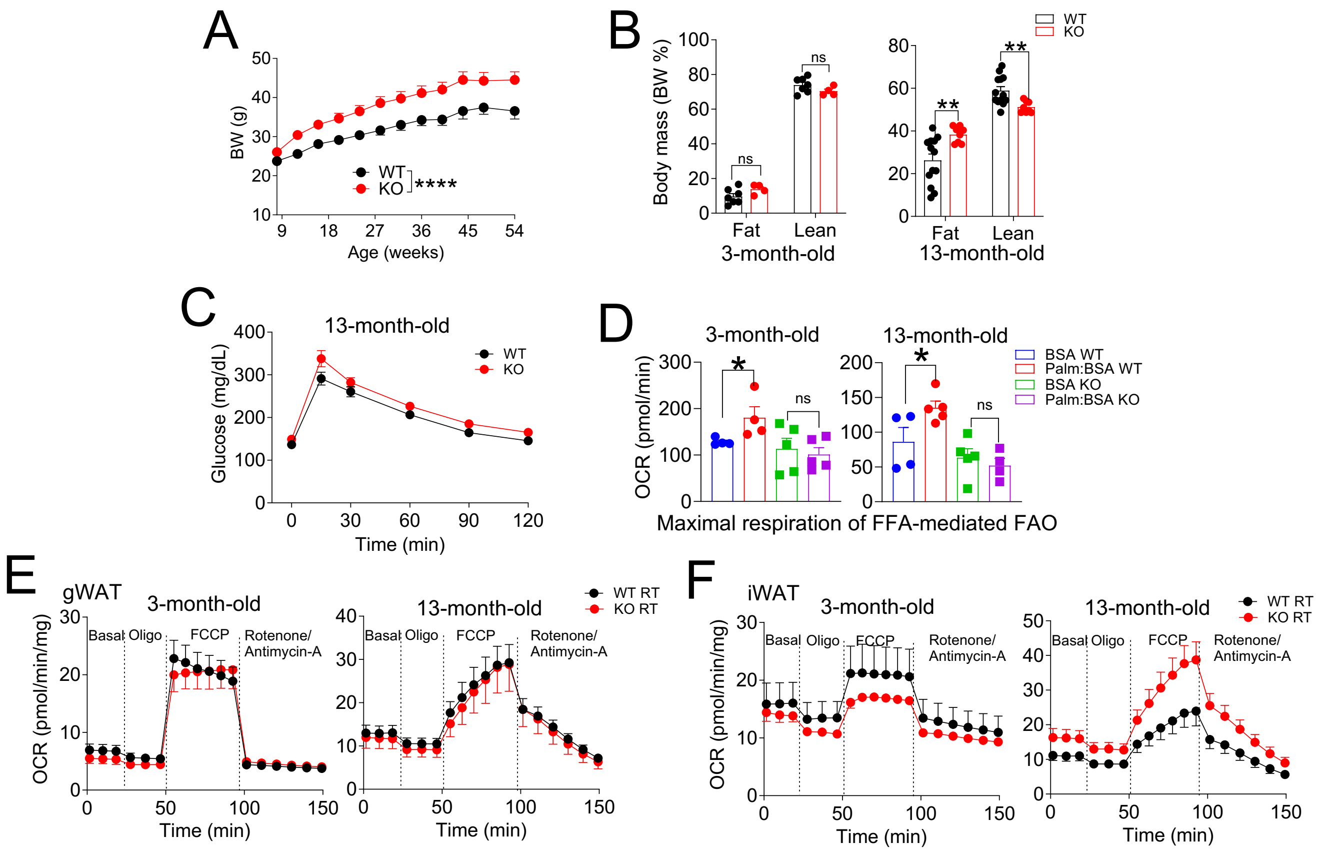

Figure S2 –Related to Figure 2. Body weight, body mass and mitochondrial function in tissues from WT and KO mice at different ages.

(A) Body weight changes of WT and KO mice. n = 10/group. **(B)** Body composition analysis including fat and lean mass for WT and GPR84 KO mice at different ages. Fat lean mass were normalized to body weight. n = 9 - 10 for old mice, n = 5 for young mice. **(C)** Glucose tolerance test of normal chow-fed aged WT and KO mice, n = 20 - 23/group. **(D)** Maximal OCR of fatty acid-dependent oxidation in BAT from WT and GPR84 KO mice at different ages. n = 5/group. **(E – F)** OCR of gWAT (**E**) and iWAT (**F**) from young and old WT and GPR84 KO mice. Data are represented as the mean ± SEM in duplicate. n = 4/group. *, P < 0.05; **, P < 0.01; ****, P<0.0001 by two-tailed Student's *t*-test for (**B**); one-tailed Student's *t*-test (**D**); two-way ANOVA followed by a Bonferroni's multiple comparison test (**A**, **E**, **F**). Data are represented as mean ± SEM.

Supplemental Figure 3. Sun *et al*. ---- related to Figure 3

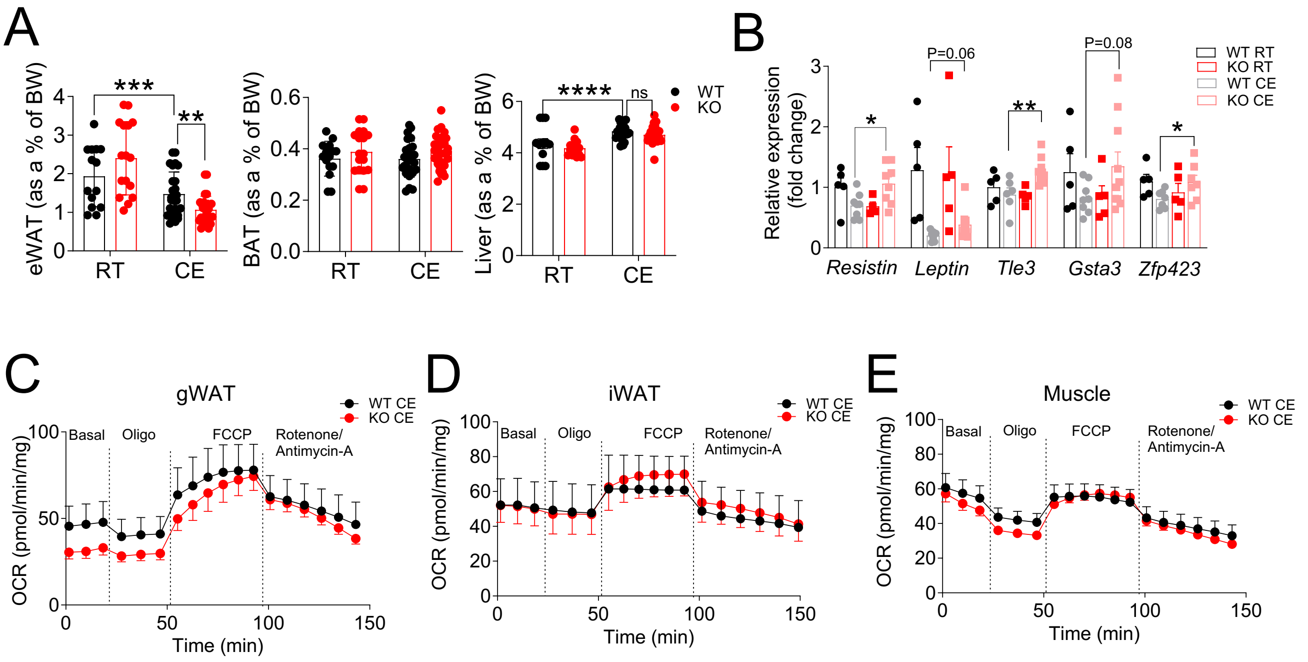

Figure S3 – Related to Figure 3. Gene expression and OCR in tissues of young WT and GPR84 KO mice at cold exposure. **(A)** Tissue weight for eWAT, BAT and liver of WT and KO mice at cold exposure. **(B)** White adipose tissue-selective gene expression in BAT of WT and KO mice at RT and cold for 6 days. n = 8-10/group. **(C – E)** OCR in gWAT (**C**), iWAT (**D**), and muscle (**E**) of mice at 6 days after cold exposure. Data are represented as mean ± SEM in duplicate. n = 5/group. *, P < 0.05; **, P < 0.01; ****, P<0.0001 by two-way ANOVA followed by a Bonferroni's multiple comparison test. Data are represented as mean ± SEM of at least three independent experiments.

Supplemental Figure 4. Sun *et al*. --- related to Figures 3 and 4

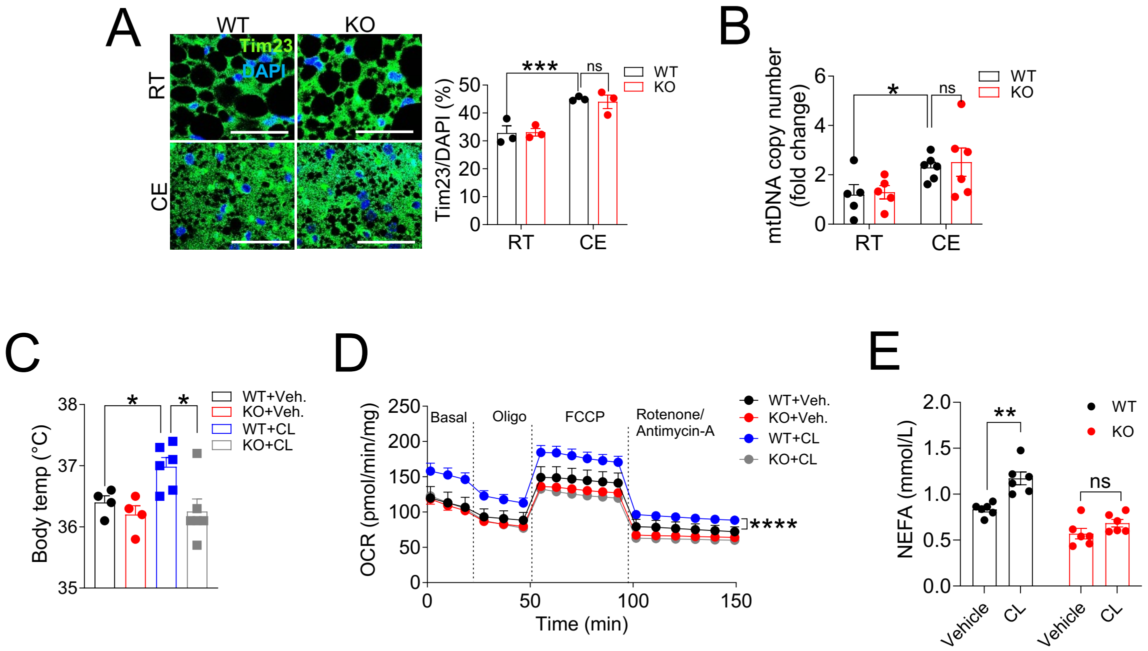

Figure S4 – Related to Figures 3 and 4. Brown adipocytes mitochondria analysis of WT and GPR84 KO mice. **(A)** Representative immunofluorescence images of Tim23 from brown adipocytes of WT and KO mice at RT and CE for 6 days. The image is a representative image from three independent experiments. n = 3/group. Scale bar = 50 μm. **(B)** The relative amount of mitochondrial DNA number was measured by qPCR from BAT of WT and KO mice at RT and CE for 6 days. Data are expressed as the mean ± SEM from more than three independent experiments. n = 8–10/group. **(C)** Body temperature of WT and KO mice was measured after vehicle (saline) or β_3 adrenergic receptor agonist CL316,243 administration for 15 minutes. n = 5 – 7/group. **(D)** OCR in BAT from WT and KO mice ± CL316,243 administration for 15 minutes. Data are represented as the mean ± SEM in duplicate. n = 3/group. **(E)** Cellular NEFA level was measured in mature WT and KO brown adipocytes ± CL316,243. n = 5/group. *, P < 0.05; **, P < 0.01; ****, P<0.0001 by two-way ANOVA followed by a Bonferroni's multiple comparison test. Data are represented as mean ± SEM of at least three independent experiments.

Supplemental Figure 5. Sun *et al*. --- related to Figure 4

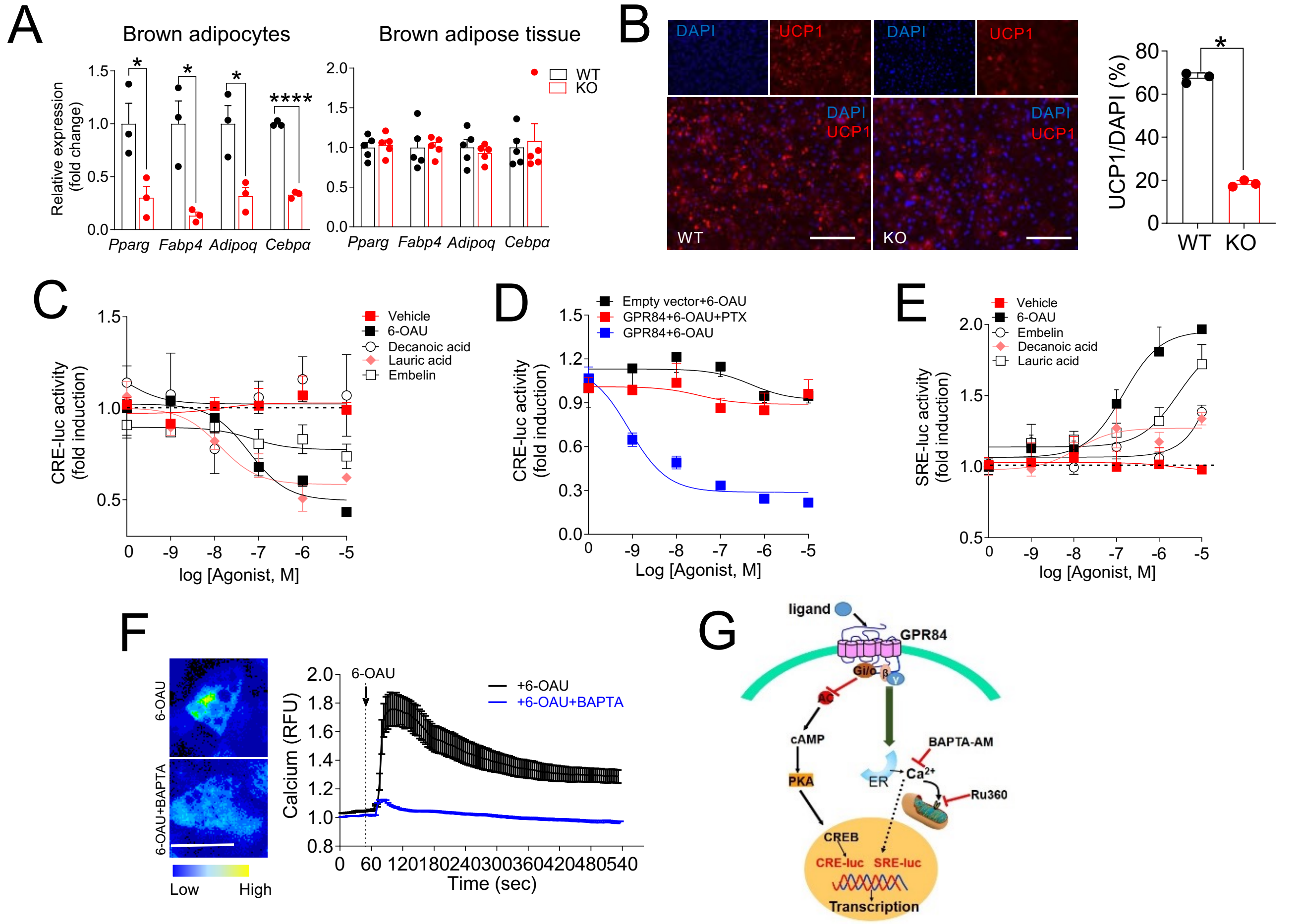

Figure S5 –Related to Figure 4. GPR84 stimulation-mediated signal transduction in brown adipocytes.
(A) Adipogenic genes were measured by qPCR in fully differentiated brown adipocytes from WT and GPR84 KO mice (left-hand side) and in BAT from WT and GPR84 KO mice (right-hand side). Data are expressed as the mean ± SEM of at least three independent experiments in triplicate. n = 3/group for brown adipocytes; n = 5/group for BAT. **(B)** Representative images of UCP1 immunofluorescence in brown adipocytes from WT and KO mice. n = 3/group. Scale bar = 50 µm. **(C)** CRE-luc activity in 293 cells transiently expressed GPR84. Cells were pre-treated with forskolin and then incubated with 6-OAU, Embelin, Decanoic acid (C10:0) and Lauric acid (C12:0) for 6h. n = 4 each concentration. **(D)** HEK293 cells transiently expressed GPR84 were pre-treated with PTX overnight and incubated with 6-OAU for 6 h. **(E)** SRE-Luc activity in 293 cells transiently expressed GPR84 were stimulated with 6-OAU, Embelin, Decanoic acid and Lauric acid for 6 h. **(F)** Calcium mobilization in mature WT brown adipocytes treated ± 6-OAU. After incubation with Fluo-4-AM for 1h RT, WT brown adipocytes ± BAPTA-AM (25 µM). Scale bar = 50 µm. Relative fluorescence units were analyzed by ROI using Image J. **(G)** Schematic diagram for GPR84-mediated signal transduction in brown adipocytes. Left-handed side cascade pathway indicates GPR84-G_i-mediated pathway while right–handed side pathway indicates the GPR84-Gβγ-mediated calcium pathway. *, $P < 0.05$; **, $P < 0.01$ by two-tailed Student's *t*-test. Data are represented as mean ± SEM from at least three independent experiment in triplicate.

Supplemental Figure 6. Sun *et al*. --- related to Figure 6

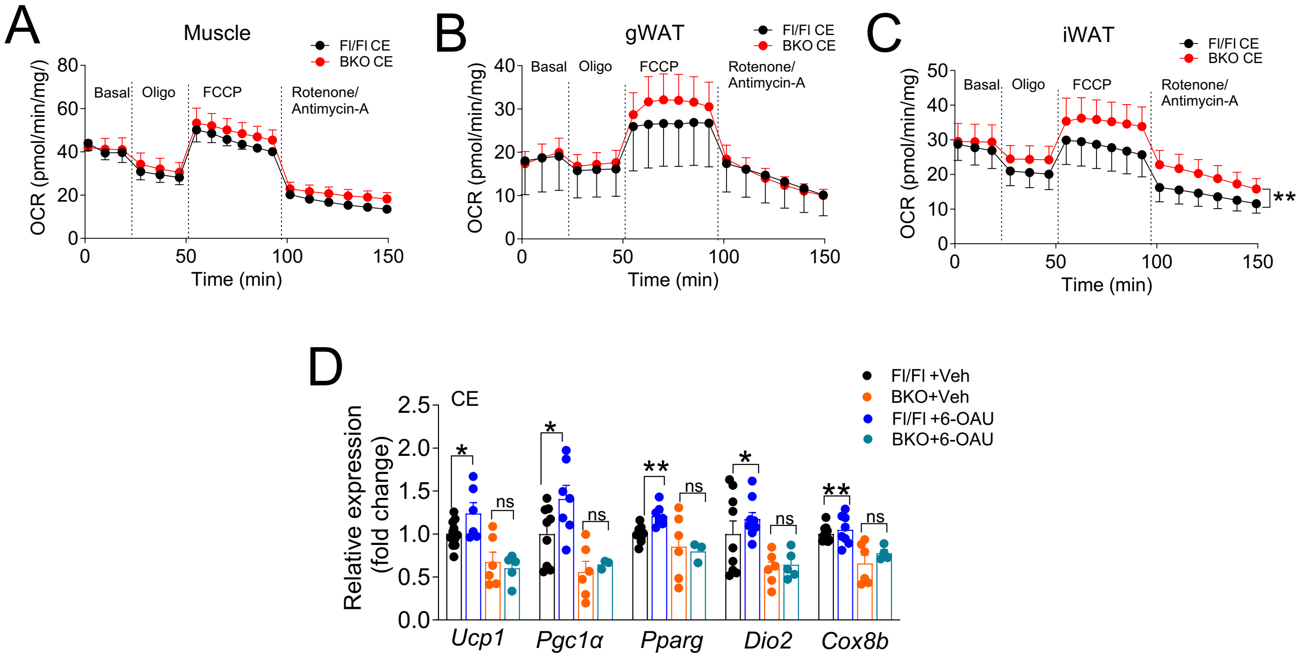

Figure S6 –Related to Figure 6. GPR84$^{fl/fl}$ and GPR84^{BKO} mice exposed to cold for 6 days.
(A–C) OCR in muscle (**A**) and gWAT (**B**) and iWAT (**C**) from GPR84$^{fl/fl}$ and GPR84^{BKO} mice at 6 days after cold exposure. The data are represented as the mean ± SEM in duplicate. n = 4/group. **(D)** Thermogenic gene expression level was measured by qPCR in BAT of vehicle- and 6-OAU-treated GPR84$^{fl/fl}$ and GPR84^{BKO} mice at 6 days after cold exposure. n = 3 – 8/group. *, P < 0.05; **, P < 0.01 by two-tailed Student's *t*-test (**D**), two-way ANOVA followed by a Bonferroni's multiple comparison test (**A**, **B**, **C**). Data are represented as mean ± SEM from at least three independent experiments.
